# Moderate Exercise during Pregnancy in Wistar Rats Alters Bone and Body Composition of the Adult Offspring in a Sex-Dependent Manner

**DOI:** 10.1371/journal.pone.0082378

**Published:** 2013-12-05

**Authors:** Brielle V. Rosa, Hugh T. Blair, Mark H. Vickers, Keren E. Dittmer, Patrick C. H. Morel, Cameron G. Knight, Elwyn C. Firth

**Affiliations:** 1 GRAVIDA: National Centre for Growth and Development, Institute of Veterinary, Animal and Biomedical Sciences, Massey University, Palmerston North, New Zealand; 2 GRAVIDA: National Centre for Growth and Development, The Liggins Institute, The University of Auckland, Auckland, New Zealand; 3 Institute of Veterinary, Animal and Biomedical Sciences, Massey University, Palmerston North, New Zealand; 4 Department of Ecosystem and Public Health, Faculty of Veterinary Medicine, University of Calgary, Calgary, Canada; The University of Manchester, United Kingdom

## Abstract

Exercise during pregnancy may have long-lasting effects on offspring health. Musculoskeletal growth and development, metabolism, and later-life disease risk can all be impacted by the maternal environment during pregnancy. The skeleton influences glucose handling through the actions of the bone-derived hormone osteocalcin. The purpose of this study was to test the effects of moderate maternal exercise during pregnancy on the bone and body composition of the offspring in adult life, and to investigate the role of osteocalcin in these effects. Groups of pregnant Wistar rats either performed bipedal standing exercise to obtain food/water throughout gestation but not lactation, or were fed conventionally. Litters were reduced to 8/dam and pups were raised to maturity under control conditions. Whole body dual-energy x-ray absorptiometry, and *ex vivo* peripheral quantitative computed tomography scans of the right tibia were performed. At study termination blood and tissue samples were collected. Serum concentrations of fully and undercarboxylated osteocalcin were measured, and the relative expression levels of osteocalcin, insulin receptor, Forkhead box transcription factor O1, and osteotesticular protein tyrosine phosphatase mRNA were quantified. Body mass did not differ between the offspring of exercised and control dams, but the male offspring of exercised dams had a greater % fat and lower % lean than controls (p=0.001 and p=0.0008, respectively). At the mid-tibial diaphysis, offspring of exercised dams had a lower volumetric bone mineral density than controls (p=0.01) and in the male offspring of exercised dams the bone: muscle relationship was fundamentally altered. Serum concentrations of undercarboxylated osteocalcin were significantly greater in the male offspring of exercised dams than in controls (p=0.02); however, the relative expression of the measured genes did not differ between groups. These results suggest that moderate exercise during pregnancy can result in lasting changes to the musculoskeletal system and adiposity in offspring, in a sex-specific manner.

## Introduction

The Developmental Origins of Health and Disease (DOHaD) paradigm suggests that the environment to which an organism is exposed during prenatal development and early life can have lasting health consequences [1]. Maternal undernutrition during pregnancy has been widely studied in relation to later-life health, and animal models used in controlled studies have confirmed the long-lasting effects of undernutrition during development. The pups of undernourished pregnant rats are hypertensive, hyperphagic, and obese in mature life [2], and both under- and over-nutrition during gestation result in earlier reproductive maturation in female rat pups [3]. However, while the effects of nutritional stress during fetal development on long-term health have been well-proven, the effects on offspring development of other environmental influences during pregnancy have not been clearly defined.

Exercise during pregnancy may also affect later offspring health. In humans, exercise during pregnancy affects fetal growth through effects on placental size and blood flow in a type-, frequency-, and intensity-dependent manner [4]. Thus mothers who begin a moderate exercise program in early pregnancy will have larger babies [5], while those who continue rigorous exercise throughout pregnancy have thinner, lighter offspring [6]. Whether these effects on birth size result in later-life health effects is not yet known, as no data are currently available on the effects of exercise during pregnancy on human offspring during adulthood. The results of studies that investigated the effects of exercise during pregnancy on childhood outcomes suggest that the children of exercising mothers are cognitively advanced relative to controls and at 5 years old are slightly lighter than, but otherwise similar to, controls in terms of physical development [7–9]. 

Research using animal models has shown a dose-response effect of exercise on uteroplacental blood flow similar to that seen in humans [10]. The effects of maternal exercise during pregnancy on birth weight in rats have also varied with the type and intensity of the exercise performed, with results ranging from no change [11–13] to significant decreases [14–16] in birth weight, the latter being associated with higher intensity maternal exercise. Studies examining the effects of exercise during pregnancy on factors other than birth weight have shown increased brain-derived neurotrophic factor and improved learning and memory in the pups of exercised rat dams relative to those of controls [11,17]. Studies examining the effects of maternal exercise during pregnancy on adult health in rats are few, but the available data suggest that maternal exercise can indeed have a long-term effect on the health of the offspring: a recent study showed that maternal treadmill running during pregnancy enhances insulin sensitivity in the adult offspring [18]. 

Although there has been great interest in the long-term metabolic effects of early life influences, the musculoskeletal system may also be affected by environmental factors during development. Recent research in mice has revealed that bone is a key regulator of metabolism, and thus changes to the skeleton may also affect metabolic health. Osteoblasts produce osteocalcin (OC), the undercarboxylated form of which can act as an endocrine hormone and regulate glucose handling by stimulating insulin production and sensitivity [19,20]. Developmental influences that alter osteoblast activity may result in changes to whole body glucose handling. It has also been suggested that the skeletal disorder osteoporosis may fit the DOHaD concept [21] and in humans there is a relationship between birth weight and adult bone mass [23]; this may reflect the associations of birth weight and infant growth with muscle size and strength in later life [24,25]. However, there has been very little research examining the effects of exercise during pregnancy on the musculoskeletal system of the offspring in adulthood. To our knowledge, the only data available on this topic are those of Monteiro et al. [22], who investigated the effects of both exercise during pregnancy and protein malnutrition during pregnancy and lactation, and found that only protein malnutrition, and not maternal exercise, affected femur length in adult offspring.

Since maternal exercise during pregnancy may have beneficial effects on the metabolism and musculoskeletal system of the offspring in later life, we hypothesized that the adult offspring of dams that exercised during pregnancy would have improved musculoskeletal and metabolic health relative to the adult offspring of control rats. Since bone and metabolism are linked by the endocrine actions of the undercarboxylated form of OC, we further surmised that serum concentrations of this bone-derived hormone would differ in the adult offspring of exercised and control dams. In this study, we utilized rising to an erect bipedal stance [23], which we have previously shown to be a non-stressful exercise for pregnant rats [13,24], to test the long-term effects of maternal exercise during pregnancy on the musculoskeletal and metabolic health of the offspring, and to investigate the role of OC in these effects.

## Materials and Methods

### Animals

Twenty virgin female Wistar rats were randomly assigned to one of two age- and weight-matched groups (exercise and control). Rats in the exercise group (DAMEX) were habituated to their bipedal stance exercise over a 5 day period, during which the height of their cages was raised incrementally each day until they had to stand on extended hindlimbs to reach the feeder and water bottle in the cage lid, and then mated. Mating took place in standard control height cages with a wire mesh floor to facilitate identification of the semen plug. Once the plug was observed the DAMEX rats were housed in raised cages throughout gestation so that they had to achieve an erect bipedal stance to obtain food and water as described previously [25]. Rats in the control group (DAMCON) were housed in cages of conventional height for the duration of the trial. Fifteen (7 control and 8 exercised) of the 20 females gave birth to litters of 8 or more live pups. Of the remaining 5 mated females, three (1 DAMCON and 2 DAMEX) did not become pregnant, and two (both DAMCON) had less than eight live pups each. A detailed description of the dam exercise regime and early-life pup outcomes from birth until weaning is provided in Rosa et al. (2012) [13].

On the day after parturition litter sizes were reduced to 8 per litter and bipedal stance exercise was stopped. All mothers and pups were then housed in control housing throughout the lactation period. Pups were weaned at lactation day 25. Male pups were pair housed from weaning until day 98 ± 2, at which time they were separated into individual cages to allow more space for their larger body size and to prevent fighting. Female pups were pair housed from weaning until study termination. All rats were housed in a climate-controlled dedicated animal facility with a 12:12 hour light:dark cycle. Feed (Research Diets AIN-93G) and water were provided *ad libitum*. Rats were bedded on kiln-dried wood shavings, and after weaning all offspring were provided with PVC tubes to allow sheltering behaviour and with marbles for enrichment.

### Ethics Statement

This study was carried out in strict accordance with the recommendations in the Guide for the Care and Use of Laboratory Animals of the National Institutes of Health. The Massey University Animal Ethics Committee approved the study protocol and all animal procedures (permit number: 10/72). All rats were anesthetized prior to imaging procedures with a mixture consisting of 0.5 mL ketamine (100 mg/mL), 0.2 mL acepromazine (2 mg/mL), 0.1 mL xylazine (100 mg/mL), and 0.2 mL sterile water injected intraperitoneally at a dose rate of 0.6 mL/100 g live weight via a 25 g needle. Every effort was made to minimize animal suffering.

### Feed Intake and Puberty Assessment

The female offspring were visually inspected once daily from day 27 until vaginal opening (indicating the onset of puberty) was observed. The rats were weighed weekly except when the daily feed intake and live weight gain of all rats was recorded over a one week period beginning when the rats were 98-101 days old; measurement of food intake was started two days after the males were moved to single housing to allow them to adjust to the cage change. Since the females were pair-housed, individual feed efficiencies were obtained for only male rats. 

### Grip Strength

Forelimb grip strength was measured using a grip strength meter for rats (Columbus Instruments, Columbus, Ohio, USA) when the rats were 167 - 172 days old. Rats were allowed to grasp a metal bar connected to the force meter and were then held at the base of the tail and pulled slowly backwards until they released their grasp. Grip strength testing was repeated 5 times with a rest period of 15-30 seconds between each test. All tests were performed by the same handler, and grip strength was defined as the mean value of all successful measurements.

### Imaging

Dual-energy X-ray absorptiometry (DXA) was used to measure bone and soft tissue parameters as described previously [25]. Male rats were scanned twice during the trial, once at 114–118 days old and a second time 2 weeks prior to euthanasia (age 187–193 days). Female rats underwent only one DXA scan at age 227–232 days; also 2 weeks prior to euthanasia. Peripheral quantitative computed tomography (pQCT) scans of the right tibia were performed *ex vivo*. Scans were made at the proximal tibial metaphysis 5 mm distal to the tibial plateau, and at the midpoint of the tibia as described previously [13]. The CV for *ex vivo* pQCT bone parameters ranged from 0.5%–2.6%. 

### Sample collection

Male rats were euthanized at 201–207 days of age, and female rats were euthanized at 242–249 days of age. The rats were fasted for 12 hours prior to euthanasia and were killed by anesthetic overdose followed by terminal blood collection as described previously [13]. All sample collection was performed between 0800 and 1200 hours to minimize the effects of circadian variation on measured parameters. Blood glucose was immediately assessed using an Accu-check Advantage blood glucose meter (Roche Diagnostics GmbH, Mannheim, Germany). Blood samples were collected into plain and Ca-ethylenediaminetetraacetic acid vacutainer tubes and immediately placed on ice; both serum and plasma were separated and stored at -80°C within one hour of collection. Immediately following euthanasia, nose-tail, head and spine length (from the occipital condyles to the palpable ischiatic tuberosity of the pelvis) were measured, and samples of bone and soft tissues were collected for future gene expression analysis; these were snap frozen in liquid nitrogen and stored at -80°C. The right tibia and both kidneys were fixed in 4% paraformaldehyde for one week and then stored in 70% ethanol. All tissue samples were collected within 20 minutes of death. Because several rats had evidence of renal pathology on post mortem examination, the kidneys of all rats were examined by a veterinary pathologist and scored from 1–4 for degree of nephropathy where 1 = 0–10% glomeruli affected, 2 = 10–40% glomeruli affected, 3 = 40–80% glomeruli affected, 4 = end-stage renal disease (80–100% glomeruli affected). Rats with nephropathy scores ≥ 3 were excluded from all post-weaning analyses.

### Osteocalcin assay

Serum levels of carboxylated (cOC) and undercarboxylated (uOC) osteocalcin were measured using commercially available, highly sensitive, rat-specific EIA kits (MK 126 and 146, Takara Bio Inc., Otsu, Japan). All samples were assayed in duplicate and the average result for each sample was used for statistical analysis. The intra-assay CVs were 5.3% and 3.5% for the cOC and uOC assays, respectively. 

### Gene Expression

At euthanasia the left femur was removed, cleaned of soft tissue, cut into equal length thirds with a small hacksaw, flushed clean of bone marrow using saline, and snap frozen in liquid nitrogen. Prior to RNA extraction the cleaned femoral diaphyses were pre-crushed using a MicroCryoCrusher (BioSpec, Oklahoma, USA) with liquid nitrogen cooling, and then 50 mg samples were homogenized by agitation in a Mini-Beadbeater-16 (BioSpec, Oklahoma, USA) in 1 ml of Tri-Reagent (T9424, Sigma-Aldrich, Auckland, New Zealand). RNA was extracted using chloroform with isopropanol precipitation and the RNA pellet resuspended in 50 µL of diethylpyrocarbonate treated water. The extracted RNA was DNase-treated with TURBO-DNA free (Ambion, Life Technologies, Texas, USA) according to the manufacturer’s instructions. RNA concentration and purity was determined using a Nanodrop ND-1000 Spectrophotometer (Thermoscientific, Wilmington, USA) and Qubit 2.0 (Life Technologies, Carlsbad, USA) followed by storage at -80°C prior to further analysis.

The RNA was converted to cDNA using the Roche Transcriptor cDNA synthesis kit (Roche Applied Science, Mannheim, Germany) as per the manufacturer’s instructions. A mix of 2.5 µM oligo(dT), 60 µM random hexamers, 1 mM each dNTP, 20 U RNase Inhibitor, 10 U reverse transcriptase, 5X reaction buffer, 250 ng RNA, and water, up to a final volume of 20 µL, was added to each tube. Samples were incubated at 25°C for 10 min, 55°C for 30 min and 85°C for 5 min. Real time quantitative polymerase chain reaction (qPCR) analyses for hydroxymethylbilane synthase (Hmbs), ß-actin (ActB), osteocalcin (OC), insulin receptor (InsR), Forkhead box transcription factor O1 (FoxO1), and osteotesticular protein tyrosine phosphatase (Esp) were performed using the StepOne Real-Time PCR system (Applied Biosystems, California, USA) and Taqman® primer/ probe sets (Applied Biosystems, Life Technologies, Texas, USA). Each qPCR reaction mix contained 1X Taqman® gene expression assay (Applied Biosystems, Life Technologies, Texas, USA), 1X Taqman® gene expression master mix (Applied Biosystems, Life Technologies, Texas, USA), 3 µL cDNA and H_2_O, up to a final volume of 10 µL. Thermal cycling conditions included an initial hold at 50°C for 2 minutes, 95°C for 10 minutes, and then 40 cycles of 95°C for 15 seconds and 60°C for 1 minute. All samples were assayed in duplicate. Standard curves were performed to determine the efficiency and R^2^ of the primer/probe combinations, which were as follows: OC (assay ID Rn00566386_g1) 95.6% efficiency, R^2^=1.00; InsR (assay ID Rn00690703_m1) 94.0% efficiency, R^2^=0.98, FoxO1 (assay ID Rn01494868_m1) 95.1% efficiency, R^2^=0.98; Esp (assay ID Rn00583620_m1) 101.6% efficiency, R^2^=0.99; Hmbs (assay ID Rn00565886_m1) 95.1% efficiency, R^2^=0.99; and ActB (assay ID Rn00667869_m1) 95.4% efficiency, R^2^=1.00. Target genes were normalized to the reference genes Hmbs and ActB. The real-time data were analysed using StepOne plus software (Applied Biosystems, Life Technologies Corp., Carlsbad CA, USA) to produce relative expression ratios.

### Statistical analysis

Offspring were excluded from analysis if they had a nephropathy score ≥3 or any health problems that might have adversely influenced growth. Several rats also died during or after anesthesia. For the statistical analysis of body weight, grip strength, and imaging data, 1–4 male offspring per dam and 2–4 female offspring per dam were used; the number of animals used in the analyses is given below each table. For serum OC testing, 1 male and 2 females per litter were randomly selected from the eligible animals, females were in any stage of their estrous cycle as this has been shown to have little effect on serum concentrations of uOC and cOC [26]. The same male and one of the females used for OC testing were selected for gene expression testing. Between-group differences in pQCT, DXA, serum OC and gene expression results were assessed using a nested model, with sex, dam exercise group, and their interaction as fixed effects and dam nested within exercise group as a random effect. Log transformation was performed prior to analysis when required to achieve a normal distribution. Day of puberty attainment and the cOC:uOC ratio were not normally distributed, even after transformation, and for these variables the differences between dam exercise groups for each sex were assessed using the non-parametric Wilcoxon test. Pearson’s correlation coefficients were determined using the residuals of the variables after fitting the linear model. All data are expressed as lsmeans ± SE unless otherwise indicated. Differences are considered significant if p≤ 0.05.

## Results

### Body composition and size

We previously reported that there were no between dam exercise-group differences in the body weights of these offspring at birth or weaning [13]. This initial lack of difference in body weight persisted throughout the study. At study termination, the weights of the DAMCON offspring versus the DAMEX offspring were 434.37 ± 47.08 g versus 421.68 ± 73.52 g (p=0.14) and 709.93 ± 55.76 g versus 699.45 ± 62.08 g (p=0.97) for female and male animals, respectively. However, body size did differ significantly between the female offspring of exercised and control dams: the mean spine length of the DAMCON female offspring was 16.54 ± 1.61 cm and of the DAMEX female offspring was 15.88 ± 0.53 cm (p=0.005); whereas the mean spine lengths of the males were 18.19 ± 0.52 and 17.99 ± 0.49 cm (p=0.25) for the DAMCON and DAMEX offspring, respectively. Analysis of the DXA scans performed 2 weeks prior to euthanasia revealed significant differences in the body composition of the male DAMEX and DAMCON offspring, but not the females; male DAMEX offspring had a greater percent body fat (p=0.001) and lesser percent lean tissue (p=0.0008) than the male DAMCON offspring (Table 1). Dam exercise group did not significantly influence whole body bone mineral content (BMC), bone mineral density, or bone area as assessed by DXA scanning. The first DXA scan (performed at 114–118 days of age in males only) showed no significant differences between the DAMEX and DAMCON offspring in bone or body composition. Spine length also did not differ between the male DAMCON and DAMEX offspring at that time.

**Table 1:** Body composition of the offspring of control and exercised dams.

	**Male**		**Female**		***P-values***		
	DAMCON	DAMEX	DAMCON	DAMEX	*Ex*	*Sex*	Ex*Sex
**% Fat**	32.48 ± 1.06	37.55 ± 1.08	35.47 ± 0.98	36.27 ± 0.93	0.25	0.41	0.04
**% Lean**	64.78 ± 1.03	59.74 ± 1.04	61.47 ± 0.94	60.51 ± 0.90	0.23	0.20	0.04

Data are lsmeans ± SE.

N = 95 offspring from 15 dams.

DAMCON = offspring of control dams, DAMEX = offspring of exercised dams, Ex = dam exercise group.

Significance of difference between % Fat in male and female offspring, p = 0.001 and 0.56, respectively.

Significance of difference between % Lean in male and female offspring, p = 0.0008 and 0.46, respectively.

### Puberty attainment and feed efficiency

The age at which the female offspring attained puberty (vaginal opening) was almost significantly different between groups (DAMCON 31.67 ± 2.04 days vs. DAMEX 30.59 ± 1.59 days, p=0.06). Individual feed intake and feed efficiency of the male rats did not differ between the DAMEX and DAMCON offspring at week 16 of life (p=0.41 and 0.42 for intake and efficiency, respectively). Since the female rats were pair-housed individual feed intake and efficiency were not measured.

### Peripheral quantitative computed tomography

The results of analysis of *ex vivo* pQCT images of the right proximal tibial metaphysis and mid-diaphysis are shown in Tables 2 and 3. At the proximal tibial metaphysis there were no significant effects of exercise on any parameters, but total BMC trended lower in both male and female DAMEX offspring than in DAMCON offspring (p=0.10). At the mid-tibial diaphysis, the DAMEX offspring had lower cortical volumetric bone mineral density (BMD_v_) than the DAMCON offspring (p=0.01) of both genders. Correcting for body weight or spine length in the model did not change the significance of the values obtained.

**Table 2:** pQCT results at the right proximal tibial metaphysis.

	**Male**		**Female**		***P-values***		
	DAMCON	DAMEX	DAMCON	DAMEX	*Ex*	*Sex*	Ex*Sex
**Total BMC (mg)**	14.59 ± 0.22	13.61 ± 0.22	11.22 ± 0.20	10.79 ± 0.23	0.10	<0.0001	0.19
**Total area (mm^2^)**	24.65 ± 0.52	22.81 ± 0.53	16.23 ± 0.48	15.85 ± 0.54	0.16	<0.0001	0.15
**Total BMD_v_ (mg/cm^3^)**	595.87 ± 8.20	596.79 ± 8.36	693.97 ± 7.50	686.21 ± 8.40	0.77	<0.0001	0.59
**Log Trabecular BMC (mg)**	0.62 ± 0.07	0.46 ± 0.07	0.44 ± 0.07	0.36 ± 0.07	0.21	0.04	0.52
**Log Trabecular area (mm^2^)**	2.39 ± 0.04	2.31 ± 0.04	1.82 ± 0.04	1.81 ± 0.04	0.42	<0.0001	0.33
**Trabecular BMD_v_ (mg/cm^3^)**	174.55 ± 9.93	158.97 ± 9.98	257.57 ± 9.14	241.93 ± 10.23	0.37	<0.0001	0.998
**Cort/subcort BMC (mg)**	12.52 ± 0.14	12.01 ± 0.14	9.60 ± 0.12	9.26 ± 0.14	0.20	<0.0001	0.53
**Cort/subcort area (mm^2^)**	8.05 ± 0.23	7.51± 0.21	7.93 ± 0.23	8.12 ± 0.21	0.11	<0.0001	0.38
**Cort/subcort BMD_v_ (mg/cm^3^)**	934.30 ± 12.22	944.82 ± 12.29	964.48 ± 11.26	972.41 ± 12.60	0.55	0.02	0.91

Data are lsmeans ± SE.

N = 94 offspring from 15 dams.

DAMCON = offspring of control dams, DAMEX = offspring of exercised dams, Ex = dam exercise group, BMC = bone mineral content, BMD_v_ = volumetric bone mineral density.

**Table 3:** pQCT results at the right mid-tibial diaphysis.

	**Male**		**Female**		***P-values***		
	DAMCON	DAMEX	DAMCON	DAMEX	*Ex*	*Sex*	Ex*Sex
**Cortical BMC (mg)**	10.24 ± 0.14	9.72 ± 0.14	7.08 ± 0.13	6.87 ± 0.14	0.15	<0.0001	0.23
**Cortical area (mm^2^)**	7.50 ± 0.10	7.20 ± 0.10	5.23 ± 0.09	5.15 ± 0.10	0.26	<0.0001	0.25
**Cortical BMD_v_ (mg/cm^3^)**	1363.96 ± 3.45	1350.17 ± 3.52	1352.29 ± 3.18	1337.33 ± 3.56	0.01	0.0004	0.86
**Endosteal circumference (mm)**	6.28 ± 0.08	6.17 ± 0.08	5.00 ± 0.07	5.07 ± 0.08	0.89	<0.0001	0.21
**Periosteal circumference (mm)**	11.56 ± 0.08	11.34 ± 0.09	9.52 ± 0.08	9.51 ± 0.09	0.47	<0.0001	0.21
**Log SSI**	2.08 ± 0.02	2.01 ± 0.02	1.52 ± 0.02	1.51 ± 0.03	0.17	<0.0001	0.33

Data are lsmeans ± SE.

N = 94 offspring from 15 dams.

DAMCON = offspring of control dams, DAMEX = offspring of exercised dams, Ex = dam exercise group, BMC = bone mineral content, BMD_v_ = volumetric bone mineral density.

Because the CT scans were done after the tibias had been cleaned of soft tissue we were unable to compare the BMC at the tibial diaphysis directly with the muscle mass in that region. Therefore, we used the DXA values for total body BMC and lean mass to examine the relationship between bone mineral content and muscle. When the head was excluded from the region of interest, the BMC: lean mass ratio of the male DAMEX offspring was significantly greater than that of the male DAMCON offspring (p=0.0003), but the relationship between BMC and lean mass did not differ between female DAMEX and DAMCON offspring (p=0.23). 

*Grip strength*

Mean forelimb grip strength differed significantly between genders (p<0.0001), but did not differ between dam exercise groups (p=0.24). The average mean grip strengths were 618.87 ± 15.02 g for females and 757.43 ±16.05 g for males. Forelimb grip strength was correlated with total BMC of the tibial diaphysis (R=0.22, p=0.03), total BMD_v_ of the tibial metaphysis (R=0.22, p=0.03), and total body lean mass (R=0.20, p=0.05). Total body lean mass was also correlated with BMC of the tibial diaphysis (R=0.53, p<0.0001).

### Serum osteocalcin

There was large variation between individuals in serum OC concentrations (Table 4), with coefficients of variation of 21.0 and 38.6% for uOC, and 20.7 and 27.8% for cOC, in female and male animals, respectively. However, dam exercise significantly affected offspring serum uOC (p=0.02), with concentrations markedly greater in the male DAMEX offspring relative to male DAMCON offspring. 

**Table 4:** Serum carboxylated and undercarboxylated osteocalcin concentrations in the offspring of exercised and control dams.

	**Male**		**Female**		***P-values***			
	DAMCON	DAMEX	DAMCON	DAMEX	*Ex*	*Sex*		Ex*Sex
**cOC (ng/mL)**	137.62 ± 13.22	152.71 ± 12.36	127.93 ± 9.85	130.30 ± 8.54	0.30	0.19		0.63
**uOC (ng/mL)**	17.86 ± 2.12	25.42 ± 1.98	17.47± 1.58	18.32 ± 1.37	0.02	0.05		0.08
**cOC + uOC (ng/mL)**	155.48 ± 14.91	178.14 ± 13.95	145.40 ± 11.12	148.61 ± 9.63	0.19	0.15		0.49
cOC:uOC*	8.77 ± 4.86	6.24 ± 1.27	7.31 ± 0.056	7.23 ± 0.86	0.15 (male)		0.30 (female)	

Data are lsmeans ± SE unless otherwise indicated.

N = 44 offspring from 15 dams (1 male, 2 female/dam except for 1 excluded female).

DAMCON = offspring of control dams, DAMEX = offspring of exercised dams, Ex = dam exercise group, cOC = fully carboxylated osteocalcin, uOC = undercarboxylated osteocalcin.

^*^ Not normally distributed therefore values shown are unadjusted means ± SD and non-parametric analysis was performed. P-values were determined by two-sided Wilcoxon test.

### Gene expression

**Figure 1:**
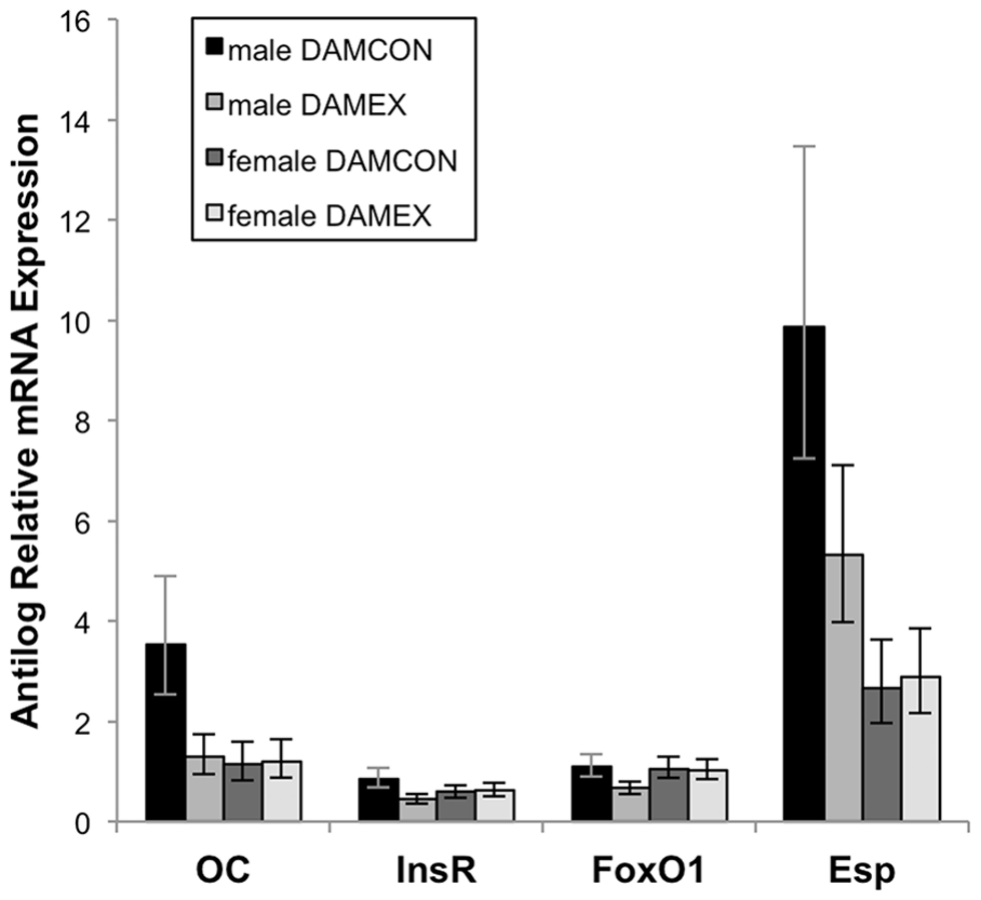
Antilog of relative expression of osteocalcin (OC), insulin receptor (InsR), Forkhead box transcription factor O1 (FoxO1), and osteotesticular protein tyrosine phosphatase (Esp) mRNA. There were no significant differences in the relative expression of these genes in the offspring of exercised and control dams. Males expressed approximately twice as much Esp mRNA as females (p=0.007). Error bars are antilog of mean ± SE on a log scale (as shown in Table 5).

**Figure 2:**
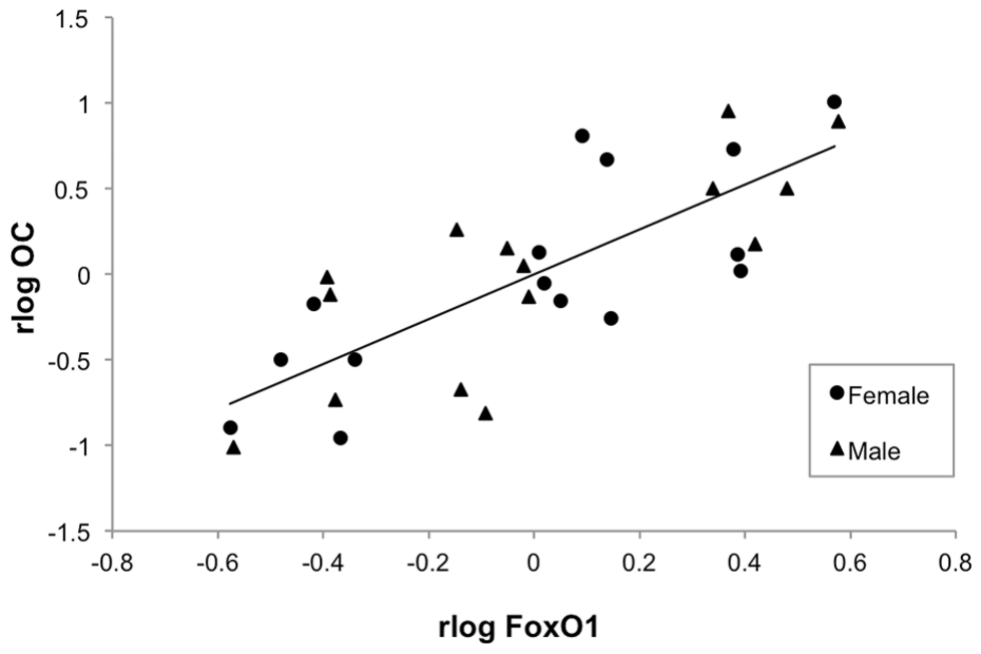
Correlation between the relative expression of Forkhead box transcription factor (FoxO1) and osteocalcin (OC) mRNA. The expression of FoxO1 and OC were significantly correlated (R=0.79, p<0.0001). rlog FoxO1 = residuals of log of FoxO1 relative expression. rlog OC = residuals of log of OC relative expression.

**Figure 3:**
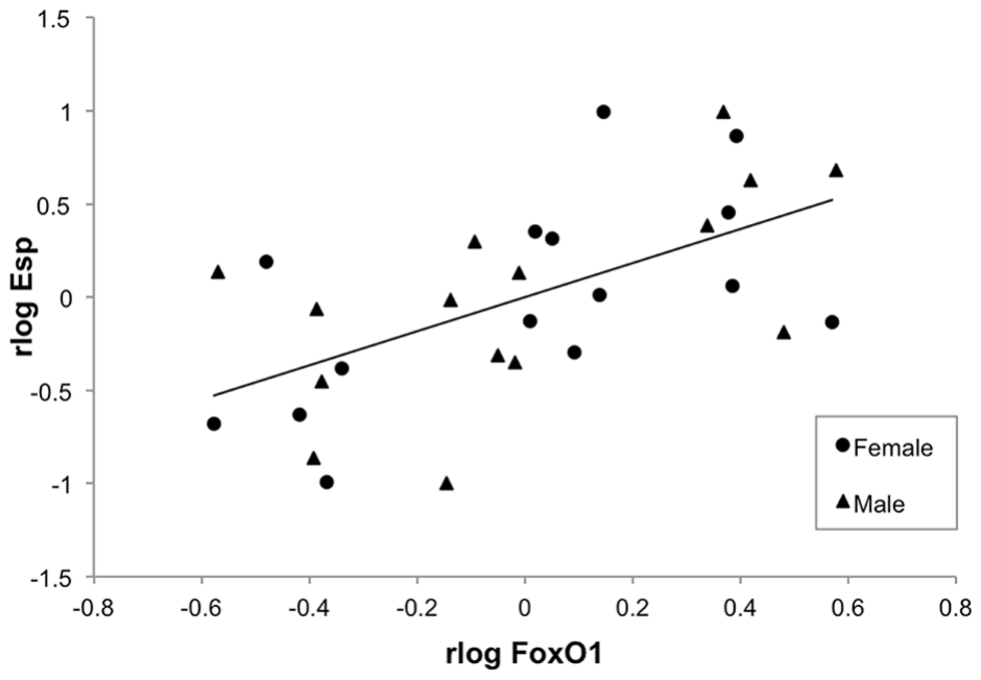
Correlation between the relative expression of Forkhead box transcription factor (FoxO1) and osteotesticular protein tyrosine phosphatase (Esp) mRNA. The expression of FoxO1 and Esp were significantly correlated (R=0.59, p=0.0006). rlog FoxO1 = residuals of log of FoxO1 relative expression. rlog Esp = residuals of log of Esp relative expression.

The log transformed relative expression levels of OC, FoxO1, InsR, and Esp mRNA are shown in Table 5 and the antilog of the mean expression levels are shown in Figure 1. Dam exercise group did not significantly affect the relative expression of these genes in either gender. Sex was a significant factor in the relative expression of Esp, with the expression levels in the males being approximately twice that in the females. In both males and females, the relative expression levels of OC and FoxO1 were highly correlated (R=0.79, p<0.0001; Figure 2), as were the relative expression levels of FoxO1 and Esp (R=0.59, p=0.0006; Figure 3). The relative expression level of InsR trended towards a correlation with the expression levels of OC (R=0.34, p=0.07), FoxO1 (R=0.34, p=0.07), and Esp (R=0.35, p=0.06), but these relationships did not reach significance. There were no significant correlations between the mean expression levels of OC, FoxO1, InsR, and Esp mRNA and serum concentrations of cOC and uOC.

**Table 5:** Log transformed relative expression levels of target genes in the offspring of exercised and control dams.

	**Male**		**Female**		***P-values***		
	DAMCON	DAMEX	DAMCON	DAMEX	*Ex*	*Sex*	Ex*Sex
**OC**	1.26 ± 0.33	0.25 ± 0.31	0.13 ± 0.33	0.18 ± 0.31	0.19	0.09	0.12
**InsR**	-0.16 ± 0.22	-0.81 ± 0.21	-0.53 ± 0.22	-0.47 ± 0.21	0.18	0.93	0.13
**FoxO1**	0.094 ± 0.20	-0.41 ± 0.19	0.053 ± 0.20	0.024 ± 0.19	0.38	0.32	0.24
**Esp**	2.29 ± 0.31	1.67 ± 0.29	0.98 ± 0.31	1.06 ± 0.29	0.33	0.0067	0.26

Data are lsmeans of log transformed relative expression levels ± SE.

N = 30 offspring from 15 dams (1 male and 1 female/dam).

DAMCON = offspring of control dams, DAMEX = offspring of exercised dams, Ex = dam exercise group, OC = osteocalcin, FoxO1 = Forkhead box transcription factor O1, InsR = insulin receptor, Esp = osteotesticular protein tyrosine phosphatase.

### Blood glucose

Blood glucose at euthanasia was lower in females than males (12.41 ± 0.25 versus 14.91 ± 0.27 g/dL, p<0.0001), but did not differ between dam exercise group in either gender (p=0.91 and 0.26 for males and females, respectively). Blood glucose concentrations were significantly correlated with percent body fat (R=0.28, p=0.007), but were not correlated with the relative expression of any of the measured genes, or any measure of serum osteocalcin.

## Discussion

To our knowledge these are the first results that demonstrate long-term effects on both body composition and bone in the offspring of dams that performed exercise during pregnancy. The long-term effects of dam exercise were evident in both the male and female offspring, but were greater in the males. Sex differences in programming effects have been demonstrated previously in both humans and animal models [27], and may be mediated by the expression of placental genes that change in response to environmental influences in both a gender- and timing-dependent manner [28]. The differences in the percentages of lean and fatty tissue in the male DAMEX and DAMCON offspring were not significant at the initial DXA scan performed at 114–118 days of age, but were highly significant by 187–193 days of age. That the lower percentage lean mass and higher percentage fat mass seen in the male DAMEX offspring are present without any differences in body weight or length, suggests that our intervention during pregnancy altered a fundamental aspect of the musculoskeletal system of the male offspring so that, when raised under control (non-exercising) conditions, the male DAMEX offspring developed less muscle than controls. This may be due to differences in their basal level of physical activity, or to an increased propensity for their stem cells to differentiate to fat instead of muscle, and may indicate a mismatch between the environment that they perceived during development and the postnatal environment that they experienced. Further research is needed to clarify the mechanisms underlying this alteration in body composition. 

Similarly, the differences in the cortical BMD_v_ of the DAMEX and DAMCON offspring suggest that the exercise performed by the dams during pregnancy resulted in a persistent change to the skeletons of their pups. At the mid-tibial diaphysis, the bones of the DAMEX offspring were both smaller in cross-sectional area and lower in BMC than the bones of the DAMCON offspring. A proportionally greater difference in between-group BMC than in bone area resulted in the significant between-group difference in BMD_v_. Both bone area and BMC change in response to the forces that act on the skeleton, and muscles are the primary source of these forces [29]. The relationship between muscle and bone can be assessed by examining the ratio of bone mineral and lean mass. In this study, the whole body BMC: lean mass ratio was greater in the male DAMEX offspring once the head, which has a much greater BMD than long bones and thus could obscure subtle whole-body changes, was excluded from the region of interest. These results indicate that the relationship between bone and muscle was fundamentally altered in the male DAMEX offspring, and that (although they had lower BMD_v_ and BMC) they had more bone mineral per gram of lean tissue than the DAMCON offspring. That such an alteration resulted from a relatively mild exposure to exercise during gestation lends support to previous reports that the bone: muscle relationship is affected by early-life influences [30].

Further evidence for long-lasting effects of early-life circumstances on the skeleton is the significant between-group difference in serum uOC concentrations, which also suggests a fundamental alteration in the bone biology of the DAMEX offspring. Osteocalcin, the most abundant non-collagenous protein produced by osteoblasts, is subject to the post-translational addition of three carboxyl groups to its amino acid chain; these increase the affinity of OC for calcium and hydroxyapatite [31]. Undercarboxylated OC lacks one, two, or all three of these carboxyl groups, and has recently been shown to act as a bone-derived hormone that regulates glucose handling (reviewed in 32). In mice, the amount of uOC released from bone depends upon the relative expression of the genes FoxO1 [33] and Esp [20,33] within the osteoblast, as well as insulin receptor activity [34], and the vitamin K status of the animal [35]. The release of uOC from bone is at least partially linked to bone resorption [36], as the acidic pH of the resorption lacuna during osteoclastic bone resorption stimulates the decarboxylation of the cOC freed from the dissolved bone matrix [34,37]. In our study, male DAMEX offspring had significantly higher uOC concentrations than male DAMCON offspring; however, their blood glucose levels did not differ. Since the expression levels of OC, FoxO1 and Esp also did not differ between groups, this suggests a difference in the balance between bone formation and resorption, with the balance shifted slightly more towards resorption in the DAMEX than the DAMCON offspring. This might be due to a mismatch between their control environment and the exercise environment that the DAMEX offspring expected.

There is now considerable evidence that uOC can act as a bone-derived endocrine hormone regulating metabolism in mice. This was first demonstrated in OC knockout mice, which develop a metabolic syndrome that includes high blood glucose, low serum insulin concentrations, and poor glucose tolerance [20]. A recent study found that injections of uOC reduced blood glucose and improved insulin sensitivity in normal mice and mice fed a high fat diet [38], providing further evidence of the important role of uOC in regulating glucose handling in the mouse. However, data from human studies have been less conclusive [39,40], and there have been no previous studies that report on the role of uOC in the metabolism of the rat. In the current study we found no correlations between blood glucose concentrations and the serum concentrations of uOC or cOC in Wistar rats. However, the studies that defined the relationship between uOC and glucose handling used knockout mice, which would have had much more severe perturbations in OC levels than our normal rats. In our animals the effects on blood glucose concentrations of changes in serum uOC or cOC levels might be too small to detect, especially considering the large individual variation in serum OC concentrations. 

We chose to investigate the expression of the OC, FoxO1, Esp, and InsR genes because FoxO1, Esp, and InsR are components of a recently described positive feedback loop that regulates the endocrine function of OC through effects on OC gene expression and carboxylation [34,41]. However, the relative expression levels of OC, FoxO1, Esp, and InsR mRNA in the mid-femur were not associated with serum cOC or uOC concentrations. There are several possible explanations for this lack of correlation. First, protein expression is determined by mRNA synthesis and degradation, and protein synthesis and degradation. A large scale study, which investigated the correlation between mRNA and protein levels *in vitro* in several thousand mouse fibroblast genes, found that mRNA levels accounted for only slightly more than 40% of the variation in protein levels [42]. Although this correlation is higher than correlations previously reported in mammalian studies [42–44], it is still low enough to suggest that regulation of protein expression is primarily post-transcriptional. Second, the lack of correlation between the expression of genes involved in OC production and regulation with serum OC concentrations in this study may also be due to the limited number and size of samples that we used for gene expression analysis. Reverse transcription qPCR was performed only on samples from the mid-femur, thus the results may not be indicative of the relative gene expression levels of the entire skeleton. Perhaps if whole skeleton expression of OC, InsR, FoxO1, and Esp were assessed, or if we had used a larger number of animals, there would have been significant correlations between expression levels and serum concentrations of cOC and uOC. However, in spite of the lack of association between gene expression and serum OC levels, the significant correlations between FoxO1 and both OC and Esp mRNA expression provides further verification of their involvement in a common pathway; as do the almost significant correlations between InsR and OC, FoxO1, and Esp mRNA expression. This, coupled with the increase in serum uOC concentrations seen in the male DAMEX offspring, suggests that the role of uOC in the metabolism of rats may be similar to its role in mice, and that it may also be similarly regulated. However, further studies examining both mRNA and protein expression are needed to clarify the role of, and regulation of, osteocalcin expression and carboxylation in rats.

Other limitations of our study include the relatively small number of dams used. Although the number of offspring used in the analyses is fairly large, the exercise intervention was performed on the dams and thus a nested analysis was used to account for the fact that the dams were the actual experimental unit. In many of the statistical analyses the effect of dam within exercise group was highly significant, indicating that it is very important to include dam as a factor when analyzing data from studies such as this one. By including dam within exercise group in our statistical models when testing whether exercise significantly affected the outcome variables, we controlled for the influence of individual dams on offspring outcomes and accounted for the hierarchical nature of our data [45]. In addition, it is important to recognize that the female offspring were approximately 40 days older than the males at the time of scanning and at sample collection. This was done for logistical reasons, but the age difference must be acknowledged when considering the effects of sex on our results. However, since all rats were over 200 days old (fully mature but not geriatric) at the time of sample collection we consider it unlikely that the difference in the ages of the male and female offspring at scanning and sample collection had a significant effect on our findings. Also of note are the number of rat offspring that were affected by nephropathy in later life. Although all of the rats appeared to be healthy at birth, by the end of the study period a total of 8 rats had significant nephropathy (histologic score ≥ 3). Of these, 6 were male, 2 were female, and all were DAMEX offspring from one of three dams. Chronic progressive nephropathy in laboratory rats is a known entity in many strains of laboratory rats, and is most commonly seen in aging males [46]. Whether the occurrence of this condition in only the DAMEX offspring in our study is related to the maternal exercise or to genetic predisposition of the dams who happened to be in the exercise group is difficult to determine with the number of animals per group that we used. Certainly nephron development occurs during gestation and the early postnatal period, and the number of nephrons that the offspring have is affected by environmental conditions during pregnancy, effects which may not be evident in altered birth weight [47]. However, most of the DAMEX offspring in our study did not have significant nephropathy, suggesting that there are other factors underlying the development of this condition. Because of the relationships between kidney disease, growth, parathyroid hormone and OC concentrations, and bone mineralization, we decided to screen all animals for histologic evidence of nephropathy and to exclude from the analysis any with a nephropathy score of ≥ 3. Thus, the animals that we have included in our analyses were physiologically “normal” to the best of our knowledge, and our results reflect the effects of maternal exercise during pregnancy on these normal offspring. Whether the maternal exercise itself predisposes the rats to develop nephropathy is a topic for future research. 

The differences that we observed between the DAMEX and DAMCON offspring were triggered by a minor intervention during development, but the actual factor or factors associated with the exercise performed by the dams in this study that resulted in these changes is difficult to determine. The increase in fatness seen in the DAMEX offspring is similar to the changes seen in the offspring of rat dams that were undernourished during pregnancy. Our exercising dams did have to stand to reach their food and water, and did have numerically (but not significantly) lower food intakes during pregnancy than did their control counterparts [13]. Perhaps this small reduction in food intake may have resulted in a subtle nutritional stress on the developing offspring that, over time, resulted in relatively mild but detectable changes in body composition. Nutritional stress during pregnancy and around the time of conception can also result in a reduced activity level in the offspring, as has been demonstrated in both rats [48] and sheep [49]. 

It is also possible that the between-group differences in our study resulted from predictive adaptive responses of the DAMEX offspring to an anticipated living situation that included exercise, and that the subsequent lack of exercise in their postnatal environment (control conditions for laboratory rats) resulted in a mismatch. A mismatch situation is one in which an organism makes predictive adaptive changes during its development in response to environmental cues, responses that would aid its survival in the environment that it expects to encounter, but then is born into an environment in which its developmental adaptations are no longer advantageous and may even be deleterious [50]. In the current study, possibly the DAMEX offspring developed anticipating a greater amount of exercise than they were allowed during postnatal life, and thus became fatter and had lower bone density than the DAMCON offspring in identical living conditions; in the latter group, the environmental conditions perceived during gestation and experienced postnatally were the same. Interpreting the differences in uOC in the male offspring, and the altered bone: muscle relationship (also more pronounced in males), is difficult at this time. Future studies that examine the effects of dam exercise during pregnancy on long-term outcomes in offspring that are also exercised postnatally will provide more insight into the specific factors underlying the effects of maternal exercise during pregnancy on adult offspring health.

In conclusion, the results of our study provide the first evidence that very moderate voluntary exercise during pregnancy can result in lasting changes to the musculoskeletal system and adiposity in the offspring without differences in birth weight. Our results provide further support to the concept of the skeleton as an organ that can be permanently altered by fetal programming, and suggest a link between uOC and metabolism in the rat.

## References

[1] Low FM, Gluckman PD, Hanson MA (2012) Developmental Plasticity, Epigenetics and Human. Health - Evol Biol 39: 650-665. doi:10.1007/s11692-011-9157-0. 21425438

[2] Vickers MH, Breier BH, Cutfield WS, Hofman PL, Gluckman PD (2000) Fetal origins of hyperphagia, obesity, and hypertension and postnatal amplification by hypercaloric nutrition. Am J Physiol Endocrinol Metab 279: E83-E87. PubMed: 10893326. 10893326 10.1152/ajpendo.2000.279.1.E83

[3] Sloboda DM, Howie GJ, Pleasants A, Gluckman PD, Vickers MH (2009) Pre- and postnatal nutritional histories influence reproductive maturation and ovarian function in the rat. PLOS ONE 4: e6744. doi:10.1371/journal.pone.0006744. PubMed: 19707592. 19707592 PMC2727050

[4] Clapp JF (2003) The effects of maternal exercise on fetal oxygenation and feto-placental growth. Eur J Obstet Gynecol Reprod Biol 110: S80-S85. doi:10.1016/S0301-2115(03)00176-3. PubMed: 12965094. 12965094

[5] Clapp JF, Kim H, Burciu B, Lopez B (2000) Beginning regular exercise in early pregnancy: Effect on fetoplacental growth. Am J Obstet Gynecol 183: 1484-1488. doi:10.1067/mob.2000.107096. PubMed: 11120515. 11120515

[6] Clapp JF, Kim H, Burciu B, Schmidt S, Petry K et al. (2002) Continuing regular exercise during pregnancy: Effect of exercise volume on fetoplacental growth. Am J Obstet Gynecol 186: 142-147. doi:10.1067/mob.2002.119109. PubMed: 11810100. 11810100

[7] Clapp JF, Simonian S, Lopez B, Appleby-Wineberg S, Harcar-Sevcik R (1998) The one-year morphometric and neurodevelopmental outcome of the offspring of women who continued to exercise regularly throughout pregnancy. Am J Obstet Gynecol 178: 594-599. doi:10.1016/S0002-9378(98)70444-2. PubMed: 9539531. 9539531

[8] Clapp JF (1996) Morphometric and neurodevelopmental outcome at age five years of the offspring of women who continued to exercise regularly throughout pregnancy. J Pediatr 129: 856-863. doi:10.1016/S0022-3476(96)70029-X. PubMed: 8969727. 8969727

[9] Pivarnik JM, Chambliss HO, Clapp JF, Dugan SA, Hatch MC et al. (2006) Impact of physical activity during pregnancy and postpartum on chronic disease risk. Med Sci Sports Exerc 38: 989-1006. doi:10.1249/01.mss.0000218147.51025.8a. PubMed: 16672855. 16672855

[10] Mottola MF (1996) The use of animal models in exercise and pregnancy research. Semin Perinatol 20: 222-231. doi:10.1016/S0146-0005(96)80015-2. PubMed: 8888448. 8888448

[11] Akhavan MM, Emami-Abarghoie M, Safari M, Sadighi-Moghaddam B, Vafaei AA et al (2008) Serotonergic and noradrenergic lesions suppress the enhancing effect of maternal exercise during pregnancy on learning and memory in rat pups. Neuroscience 151: 1173-1183. 18207332 10.1016/j.neuroscience.2007.10.051

[12] Mottola M, Bagnall KM, McFadden KD (1983) The effects of maternal exercise on developing rat fetuses. Br J Sports Med 17: 117-121. doi:10.1136/bjsm.17.2.117. PubMed: 6883019. 6883019 PMC1859029

[13] Rosa BV, Blair HT, Vickers MH, Morel PCH, Cockrem JF et al. (2012) Voluntary exercise in pregnant rats improves post-lactation maternal bone parameters but does not affect offspring outcomes in early life. J Musculoskelet Neuronal Interact 12: 199-208. PubMed: 23196262. 23196262

[14] Denadai BS, Picarro Ida C, Madjian S, Bergamaschi CT, Santos VC et al. (1994) High intensity exercise during pregnancy of rats. Effects on mother and offspring. Comp Biochem Physiol A Physiol 109: 727-740. doi:10.1016/0300-9629(94)90216-X. PubMed: 8529013. 8529013

[15] Houghton PE, Mottola MF, Plust JH, Schachter CL (2000) Effect of maternal exercise on fetal and placental glycogen storage in the mature rat. Can J Appl Physiol 25: 443-452. doi:10.1139/h00-029. PubMed: 11098156. 11098156

[16] Treadway J, Dover EV, Morse W, Newcomer L, Craig BW (1986) Influence of exercise training on maternal and fetal morphological characteristics in the rat. Journal of Appl Physiol 60: 1700-1703. PubMed: 3710987. 3710987 10.1152/jappl.1986.60.5.1700

[17] Lee H-H, Kim H, Lee J-W, Kim Y-S, Yang H-Y et al. (2006) Maternal swimming during pregnancy enhances short-term memory and neurogenesis in the hippocampus of rat pups. Brain Dev 28: 147-154. doi:10.1016/j.braindev.2005.05.007. PubMed: 16368211. 16368211

[18] Carter LG, Qi NR, De Cabo R, Pearson KJ (2013) Maternal exercise improves insulin sensitivity in mature rat offspring. Med Sci Sports Exerc 45: 832-840. doi:10.1249/MSS.0b013e31827de953. PubMed: 23247711. 23247711 PMC3617068

[19] Confavreux CB, Levine RL, Karsenty G (2009) A paradigm of integrative physiology, the crosstalk between bone and energy metabolisms. Mol Cell Endocrinol 310: 21-29. doi:10.1016/j.mce.2009.04.004. PubMed: 19376193. 19376193 PMC3667507

[20] Lee NK, Sowa H, Hinoi E, Ferron M, Ahn JD et al. (2007) Endocrine regulation of energy metabolism by the skeleton. Cell 130: 456-469. doi:10.1016/j.cell.2007.05.047. PubMed: 17693256. 17693256 PMC2013746

[21] Cooper C, Westlake S, Harvey N, Javaid K, Dennison E et al. (2006) Review: developmental origins of osteoporotic fracture. Osteoporos Int 17: 337-347. doi:10.1007/s00198-005-2039-5. PubMed: 16331359. 16331359

[22] Monteiro ACT, Paes ST, dos Santos JA, de Lira KDS, de Moraes SRA (2010) Effects of physical exercise during pregnancy and protein malnutrition during pregnancy and lactation on the development and growth of the offspring's femur. J Pediatr (Rio J) 86: 233-238. doi:10.1590/S0021-75572010000300012. PubMed: 20440444. 20440444

[23] Yao W, Jee WS, Chen JL, Li CY, Frost HM (2001) A novel method to 'exercise' rats: making rats rise to erect bipedal stance for feeding - raised cage model. J Musculoskelet Neuronal Interact 1: 241-247. PubMed: 15758498. 15758498

[24] Rosa BV, Firth EC, Blair HT, Vickers MH, Morel PCH (2011) Voluntary exercise in pregnant rats positively influences fetal growth without initiating a maternal physiological stress response. Am J Physiol Regul Integr Comp Physiol 300: R1134-R1141. doi:10.1152/ajpregu.00683.2010. PubMed: 21307360. 21307360

[25] Rosa BV, Firth EC, Blair HT, Vickers MH, Morel PCH et al. (2010) Short-term voluntary exercise in the rat causes bone modeling without initiating a physiological stress response. Am J Physiol Regul Integr Comp Physiol 299: R1037-R1043. doi:10.1152/ajpregu.00112.2010. PubMed: 20668232. 20668232

[26] Rosa BV, Blair HT, Vickers MH, Knight CG, Morel PCH et al. (2013) Serum concentrations of fully and undercarboxylated osteocalcin do not vary between estrous cycle stages in Sprague-Dawley rats. Endocrine. doi:10.1007/s12020-013-0008-x. 23817840

[27] Gilbert JS, Nijland MJ (2008) Sex differences in the developmental origins of hypertension and cardiorenal disease. Am J Physiol Regul Integr Comp Physiol 295: R1941-R1952. doi:10.1152/ajpregu.90724.2008. PubMed: 18971349. 18971349 PMC2685301

[28] Gabory A, Ferry L, Fajardy I, Jouneau L, Gothié JD et al. (2012) Maternal diets trigger sex-specific divergent trajectories of gene expression and epigenetic systems in mouse placenta. PLOS ONE 7: e47986 PubMed: 23144842. 23144842 10.1371/journal.pone.0047986 PMC3489896

[29] Frost HM (2001) From Wolff's law to the Utah paradigm: insights about bone physiology and its clinical applications. Anat Rec 262: 398-419. doi:10.1002/ar.1049. PubMed: 11275971. 11275971

[30] Firth EC, Rogers CW, Vickers M, Kenyon PR, Jenkinson CM et al. (2008) The bone-muscle ratio of fetal lambs is affected more by maternal nutrition during pregnancy than by maternal size. Am J Physiol Regul Integr Comp Physiol 294: R1890-R1894. doi:10.1152/ajpregu.00805.2007. PubMed: 18385462. 18385462

[31] Lee AJ, Hodges S, Eastell R (2000) Measurement of osteocalcin. Ann Clin Biochem 37: 432-446. doi:10.1258/0004563001899573. PubMed: 10902858. 10902858

[32] Ducy P (2011) The role of osteocalcin in the endocrine cross-talk between bone remodelling and energy metabolism. Diabetologia 54: 1291-1297. doi:10.1007/s00125-011-2155-z. PubMed: 21503740. 21503740

[33] Rached MT, Kode A, Silva BC, Jung DY, Gray S et al. (2010) FoxO1 expression in osteoblasts regulates glucose homeostasis through regulation of osteocalcin in mice. J Clin Invest 120: 357-368. doi:10.1172/JCI39901. PubMed: 20038793. 20038793 PMC2798687

[34] Ferron M, Wei JW, Yoshizawa T, Del Fattore A, DePinho RA et al. (2010) Insulin signaling in osteoblasts integrates bone remodeling and energy metabolism. Cell 142: 296-308. doi:10.1016/j.cell.2010.06.003. PubMed: 20655470. 20655470 PMC2910411

[35] Vermeer C, Gijsbers B, Craciun AM, GroenenvanDooren M, Knapen MHJ (1996) Effects of vitamin K on bone mass and bone metabolism. J Nutr 126: S1187-S1191. 10.1093/jn/126.suppl_4.1187S 8642454

[36] Ivaska KK, Hentunen TA, Vääräniemi J, Ylipahkala H, Pettersson K et al. (2004) Release of intact and fragmented osteocalcin molecules from bone matrix during bone resorption in vitro. J Biol Chem 279: 18361-18369. doi:10.1074/jbc.M314324200. PubMed: 14970229. 14970229

[37] Clemens TL, Karsenty G (2011) The osteoblast: An insulin target cell controlling glucose homeostasis. J Bone Miner Res 26: 677-680. doi:10.1002/jbmr.321. PubMed: 21433069. 21433069

[38] Ferron M, McKee MD, Levine RL, Ducy P, Karsenty G (2012) Intermittent injections of osteocalcin improve glucose metabolism and prevent type 2 diabetes in mice. Bone 50: 568-575. doi:10.1016/j.bone.2011.04.017. PubMed: 21550430. 21550430 PMC3181267

[39] Motyl KJ, McCabe LR, Schwartz AV (2010) Bone and glucose metabolism: A two-way street. Arch Biochem Biophys 503: 2-10. doi:10.1016/j.abb.2010.07.030. PubMed: 20682281. 20682281 PMC2946845

[40] Booth SL, Centi A, Smith SR, Gundberg C (2013) The role of osteocalcin in human glucose metabolism: marker or mediator? Nat Rev Endocrinol 9: 43-55. PubMed: 23147574. 23147574 10.1038/nrendo.2012.201 PMC4441272

[41] Karsenty G, Oury F (2012) Biology Without Walls: The Novel Endocrinology of Bone. In: Julius D Clapham DE Annu Rev Physiol 74: 87-105. 22077214 10.1146/annurev-physiol-020911-153233 PMC9277655

[42] Schwanhäusser B, Busse D, Li N, Dittmar G, Schuchhardt J et al. (2011) Global quantification of mammalian gene expression control. Nature 473: 337-342. doi:10.1038/nature10098. PubMed: 21593866. 21593866

[43] Tian Q, Stepaniants SB, Mao M, Weng L, Feetham MC et al. (2004) Integrated genomic and proteomic analyses of gene expression in mammalian cells. Mol Cell Proteomics 3: 960-969. PubMed: 15238602. 15238602 10.1074/mcp.M400055-MCP200

[44] Maier T, Güell M, Serrano L (2009) Correlation of mRNA and protein in complex biological samples. FEBS Lett 583: 3966-3973. doi:10.1016/j.febslet.2009.10.036. PubMed: 19850042. 19850042

[45] Kwong WY, Osmond C, Fleming TP (2004) Support for Barker hypothesis upheld in rat model of maternal undernutrition during the preimplantation period: application of integrated 'random effects'. Statistical Model - Reprod Biomed Online 8: 574-576. doi:10.1016/S1472-6483(10)61105-4. 15151724

[46] Hard GC, Khan KN (2004) A contemporary overview of chronic progressive nephropathy in the laboratory rat, and its significance for human risk assessment. Toxicol Pathol 32: 171-180. doi:10.1080/01926230490422574. PubMed: 15200155. 15200155

[47] Moritz KM, Singh RR, Probyn ME, Denton KM (2009) Developmental programming of a reduced nephron endowment: more than just a baby's birth weight. Am J Physiol Renal Physiol 296: F1-F9. PubMed: 18653482. 18653482 10.1152/ajprenal.00049.2008

[48] Vickers MH, Breier BH, McCarthy D, Gluckman PD (2003) Sedentary behavior during postnatal life is determined by the prenatal environment and exacerbated by postnatal hypercaloric nutrition. Am J Physiol Regul Integr Comp Physiol 285: R271-R273. PubMed: 12794001. 12794001 10.1152/ajpregu.00051.2003

[49] Donovan EL, Hernandez CE, Matthews LR, Oliver MH, Jaquiery AL et al. (2013) Periconceptional undernutrition in sheep leads to decreased locomotor activity in a natural environment. J Dohad 4: 296-299. 10.1017/S2040174413000214 24993003

[50] Gluckman P, Hanson M (2006) Mismatch: How Our World No Longer Fits Our Bodies. Oxford: Oxford University Press.

